# Coping with rheumatic stressors in long-standing axial spondyloarthritis: association with patient and disease characteristics

**DOI:** 10.1093/rheumatology/keag140

**Published:** 2026-03-25

**Authors:** Marc van Essen, Dafne Capelusnik, Désirée van der Heijde, Robert B M Landewé, Wim van Lankveld, Astrid van Tubergen, Sofia Ramiro, Annelies Boonen

**Affiliations:** Faculty of Health, Medicine and Life Sciences, Maastricht University, Maastricht, The Netherlands; Care and Public Health Research Institute (CAPHRI), Maastricht University, Maastricht, The Netherlands; Department of Rheumatology, Tel Aviv Sourasky Medical Center, Tel Aviv, Israel; Department of Rheumatology, Leiden University Medical Center, Leiden, The Netherlands; Department of Rheumatology & Clinical Immunology, Amsterdam University Medical Centers, University of Amsterdam, Amsterdam, The Netherlands; Department of Rheumatology, Zuyderland Medical Center, Heerlen, The Netherlands; Musculoskeletal Rehabilitation Research Group, Institute of Health Studies, HAN University of Applied Sciences, Nijmegen, The Netherlands; Care and Public Health Research Institute (CAPHRI), Maastricht University, Maastricht, The Netherlands; Department of Rheumatology, Maastricht University Medical Center, Maastricht, The Netherlands; Department of Rheumatology, Leiden University Medical Center, Leiden, The Netherlands; Department of Rheumatology, Zuyderland Medical Center, Heerlen, The Netherlands; Care and Public Health Research Institute (CAPHRI), Maastricht University, Maastricht, The Netherlands; Department of Rheumatology, Maastricht University Medical Center, Maastricht, The Netherlands

**Keywords:** axial spondyloarthritis, coping strategies, epidemiology, longitudinal studies

## Abstract

**Objectives:**

Coping strategies have been shown to influence health outcomes of persons with axSpA. This study explores which stable and variable factors are associated with coping and whether coping strategies change over time.

**Methods:**

Data from the Outcome in AS International Study (OASIS) were used. Coping was assessed at year four and six of follow-up with the COping with Rheumatic Stressors (CORS) questionnaire, which assesses coping with pain, limitations and dependence across eight strategies (score 1–4). Factors associated with each strategy, including stable patient characteristics and variable axSpA health scores, were analysed with multivariable Generalized Estimation Equations (GEE). Separate GEE analyses assessed changes in coping strategies over time.

**Results:**

A total of 116 patients were included. Of the eight strategies, *optimism* [mean: 3.1 (SD: 0.5)], *comforting cognitions* [3.0 (0.5)] and *showing consideration* [2.8 (0.4)] were the most used. Among stable socio-demographic and disease characteristics, education showed the most prominent associations with higher education being linked to more use of *comforting cognitions* [β: −0.22 (95%CI: −0.41; −0.03)] and *optimism* [−0.28 (−0.49; −0.07)]. Among variable health scores, higher Short Form 36 Mental Component Scores were very weakly associated with *comforting cognitions* [0.01 (0.00; 0.02)] and *optimism* [0.01 (0.00; 0.02)], while fatigue (BASDAI Q1) was associated with *decreasing activities* [0.04 (0.00; 0.09)]. Coping strategies did not change over time (range β_time_: −0.04–0.07/two years).

**Conclusion:**

Coping strategies in axSpA are more strongly influenced by stable personal traits than by variable health scores and remain stable over time, suggesting coping reflects individual disposition rather than disease state.

Rheumatology key messages

- In axial spondyloarthritis, comforting cognitions, optimism and showing consideration were the most used coping strategies.
- The use of coping strategies does not change over time.
- Coping depends more on stable characteristics (e.g. education) than variable health scores (e.g. disease activity).

## Introduction

Axial spondyloarthritis (axSpA) is a chronic inflammatory disease primarily affecting the spine and sacroiliac joints [1, 2]. It is characterized by inflammatory back pain, stiffness, extra-musculoskeletal manifestations (EMM) and fatigue [1, 2]. AxSpA is associated with a substantial physical and social burden for patients, which can have a profound impact on a patient’s day-to-day life and work [3]. The disease often begins in early adulthood with periods of exacerbation and remission [4]. The disease burden extends beyond physical symptoms, as living with axSpA often requires patients to adapt psychologically and behaviourally [4, 5].

To manage the ongoing physical and emotional challenges or distress associated with axSpA, patients engage in different coping strategies. Coping strategies refer to efforts used to manage stressful situations. Specifically in inflammatory rheumatic diseases such as axSpA, distress is mainly related to pain, limitations and dependency [6]. Within the coping framework, cognitive and behavioural strategies are traditionally distinguished [6]. For example, pacing or decreasing activities are observable actions taken to manage symptoms and would be referred to as behavioural strategies [6]. In contrast, optimism or comforting cognitions involve internal psychological responses regarding the impact of a disease and would be referred to as cognitive strategies [6]. To explain coping strategies, two opposing theories emerged. The dispositional model assumes a consistent preference for certain coping strategies, while the situational model views coping as a dynamic and evolving process [7, 8]. According to Leventhal’s Common-Sense Model, coping strategies stem from the perceptions that individuals have towards their disease [9]. The way one person responds to a period of disease exacerbation or remission may differ substantially from another, as these responses are influenced by each individual’s unique beliefs, experiences and perceptions related to the disease [9]. This highlights the importance of considering both situational as well as dispositional factors to understand coping. While the dispositional approach reflects enduring habits or personal traits, the situational approach is shaped by characteristics of the specific situation at hand [7, 8]. Understanding how individuals cope is crucial, as coping plays an important role in shaping health outcomes.

Despite the relevance of coping, only a limited number of studies have explored coping strategies in axSpA. Most existing work is cross-sectional and the majority of these studies have focused on how coping strategies influence specific outcomes such as workforce withdrawal, health-related quality of life or physical activity [10–14]. Less is known about which factors determine the use of specific coping strategies in individuals with axSpA [14, 15]. Thus, it remains unclear whether the use of a specific coping strategy depends more on stable (dispositional) factors such as socio-demographic background, as opposed to variable (situational) factors such as fluctuations in disease and whether coping changes over time, especially in patients with long-standing disease [15, 16]. These gaps limit the ability to understand how coping develops and how it can be modified.

Identifying which patients are more likely to use certain coping strategies could support the development of personalized, targeted psychosocial or behavioural treatments. If coping strategies are modifiable, this might improve health outcomes. The present study therefore aimed to (i) explore the role of stable patient characteristics as opposed to variable health scores on the use of coping strategies in patients with axSpA and (ii) assess whether coping strategies change over two years in patients with long-standing axSpA.

## Methods

### Study population and setting

For this study, data from the Outcome in Ankylosing Spondylitis International Study (OASIS) were used. OASIS is a longitudinal, observational, multicentre study initiated in 1996 [17]. The study includes a cohort of 217 consecutive patients diagnosed with axSpA from the Netherlands, France and Belgium. All patients had to meet the modified New York criteria. Patients received standard care, prescribed at the discretion of the rheumatologist and comprising both pharmacological and non-pharmacological treatments. Pharmacological management included non-steroidal anti-inflammatory drugs (NSAIDs), conventional synthetic disease-modifying antirheumatic drugs (csDMARDs), analgesics and biologic DMARDs (bDMARDs), the latter only being prescribed infrequently when indicated from 2002 onwards. Non-pharmacological management involved exercises and physiotherapy.

Baseline data collection included information on patient characteristics, axSpA phenotypes and axSpA health scores. Follow-up visits were conducted every six months during the first two years and annually thereafter for 12 years. The coping questionnaire was first administered at the four-year evaluation (initial assessment) and again at the six-year evaluation (two-year follow-up). Patients were included in the analysis if they had data on coping at one or both timepoints.

All patients provided written informed consent. The study was approved by the ethics committees of all participating hospitals. This approval extended to the current analysis.

### Coping strategies

Coping strategies were assessed using the COping with Rheumatic Stressors (CORS) questionnaire. The CORS was developed with strong involvement of patients and aimed to measure the range of cognitive and behavioural strategies to cope with stressors specific to rheumatic diseases [6, 18]. Originally developed and validated for rheumatoid arthritis, the CORS has since been validated in axSpA and in several languages [6, 15, 18–22].

The questionnaire consists of eight coping strategies across the three stressors. Coping with pain is assessed through *comforting cognitions* (nine items), *decreasing activities* (eight items) and *diverting attention* (eight items), coping with limitations through *optimism* (five items), *pacing* (10 items) and *creative solution seeking* (eight items) and coping with dependence through *accepting dependence* (six items) and *showing consideration* (seven items). The subscales “decreasing activities” and “pacing” represent behavioural coping strategies, while the remaining six subscales represent cognitive coping strategies. Example items from each of the subscales can be seen in Supplementary Table S1. Each item is rated on a four-point Likert scale ranging from 1 (seldom or never used) to 4 (very often used), with higher scores indicating more frequent use of a particular coping strategy. Mean scores were calculated for each subscale. If more than one item was missing, the subscale score was considered to be missing. In the development study, the CORS has demonstrated good reliability (Cronbach’s α: 0.79–0.88; Pearson’s test–retest coefficient: 0.79–0.91) [23].

### Potential factors associated with coping strategies

Stable socio-demographic factors included age, sex, education level and employment status. Age at year four of the cohort was calculated based on date of birth. Sex addressed biological sex (male or female). Educational level was categorized as lower or higher, with higher education corresponding to the attainment of a bachelor’s or master’s degree. Employment status was defined as being employed [working (≥33 h per week) *vs* not working (<33 h per week)]. Stable general health/axSpA characteristics included body mass index (BMI), presence of comorbidities [rheumatic disease comorbidity index (RDCI), 0–9], disease duration and stable axSpA phenotypical characteristics {HLA-B27 status, physician-diagnosed presence of inflammatory bowel disease (IBD), uveitis or psoriasis before or at baseline of the cohort or follow-up and presence of peripheral arthritis (≥ one swollen joint) and enthesitis [Mander Enthesitis Index (MEI), 0–90]} [24]. Variable axSpA health scores comprised: disease activity [axial spondyloarthritis disease activity score (ASDAS)], physical function [Bath ankylosing spondylitis functional index (BASFI), 0–10], mobility [Bath ankylosing spondylitis metrology index (BASMI), 0–10], fatigue [Bath ankylosing spondylitis disease activity index (BASDAI) Q1, 0–10], mental health related quality of life [Short form 36 mental component score (SF36-MCS), 0–100], back pain (BASDAI Q2, 0–10), patient global assessment of disease activity (0–10) and morning stiffness (mean BASDAI Q5 and Q6, 0–10).

### Statistical analysis

Descriptive analysis was used to characterize the population selected for this analysis. To gain insight into selective drop-out, a comparison was made with patients from the OASIS population not included in the current analyses, while restricting this comparison to the baseline visit of the OASIS cohort. Correlations between coping strategies were assessed using Pearson’s correlation coefficients to determine whether strategies represented independent constructs or exhibited overlap (r > 0.50 and r<−0.50 indicating moderate overlap) [25].

Factors associated with each of the eight coping strategies were assessed with separate generalized estimating equations (GEE), using the ‘exchangeable’ correlation structure. Robust standard errors were used to allow for more reliable confidence intervals (CI) [26]. Data from both timepoints were used and observations were clustered in cases. Three distinct models were built. Model A included stable (socio-demographic and general health/axSpA) characteristics, while Models B and C additionally included variable axSpA health scores (ASDAS being replaced by its components in Model C).

When exploring the role of more stable factors (Model A), variables with *P* < 0.20 in univariable analyses were added to age (continuous), sex and education (main independent variables of interest) using manual forward selection. These (stable) factors comprised other socio-demographic factors (employment status), BMI, comorbidities and axSpA phenotype characteristics. Collinearity was checked for, and relevant interactions with the core variables of interest were explored. If an interaction was statistically significant, the stratified model was presented if clinically relevant. If not, the variable was tested as a potential confounder. If a coefficient of the association of primary interest (age, sex and education level) changed >10% upon inclusion of a new variable, this new variable was considered a relevant confounder and kept in the model. When adding the variable scores for the health outcomes, two models were built: Model B (the main axSpA health scores model) included fatigue (BASDAI Q1), BASFI, BASMI, SF36-MCS and ASDAS, whereas Model C (the alternative axSpA health scores model) excluded the ASDAS and instead incorporated its components, namely back pain (BASDAI Q2) and morning stiffness (BASDAI Q56), together with the patient’s global assessment of disease activity. Having two different models with axSpA health scores allowed for a more robust analysis of whether health scores determined coping use while not compromising collinearity between the ASDAS and BASDAI questions.

Additional GEE analyses were used to assess the potential change over time in each of the eight coping strategies. Time was analysed as the independent variable. The estimate obtained reflected the change in the use of the respective coping strategy over 2 years. Only participants with data on both timepoints for each coping strategy were included in this analysis to ensure that differences were not attributable to dropouts.

For all analyses, a *P*-value < 0.05 was considered significant. All statistics were performed with SPSS v.28.0 (IBM, Armonk, NY, USA).

## Results

In total, 116 patients [72% male, mean age 48 years (SD 12)] had valid CORS data at one or both timepoints (Table 1). Compared with the OASIS patients not included in these analyses (*n* = 101), patients in this study were less often highly educated (18% compared with 36%) and less often worked full-time (42% compared with 55%) (Supplementary Table S2).

**Table 1:** Characteristics of the study population at the initial assessment of coping.

Assessment	N = 116
Age, years, mean (SD)	49.4 (11.6)
Sex, male, *n* (%)	84 (72%)
Education level	
Lower, *n* (%)	94 82%)^a^
Higher, *n* (%)	21 (18%) ^a^
BMI, kg/m^2^, mean (SD)	26.5 (4.9)
RDCI, 0–9, mean (SD)	0.3 (0.8)
Currently working, >33 hr/week, *n* (%)	48 (42%)^a^
Duration of symptoms, years, mean (SD)	27.7 (11.6)^b^
Presence of IBD, ever, *n* (%)	14 (12%)^a^
Presence of peripheral arthritis, current, *n* (%)	12 (10%)
Presence of uveitis, ever, *n* (%)	29 (25%)^a^
Presence of psoriasis, ever, *n* (%)	5 (4%)^a^
Enthesitis (MEI), 0–90, mean (SD)	9.8 (16.0)^b^
HLA-B27 positive, *n* (%)	89 (80%)^b^
ASDAS, mean (SD)	2.6 (1.0)^c^
BASFI, 0–10, mean (SD)	3.9 (2.6)^b^
BASMI, 0–10, mean (SD)	3.9 (1.7)^c^
BASDAI Q1 (fatigue), 0–10, mean (SD)	4.5 (2.8)
SF36 MCS, 0–100, mean (SD)	51.0 (11.9)^a^
BASDAI Q2 (back pain), 0–10, mean (SD)	4.2 (2.7)
Patient global assessment, 0–10, mean (SD)	3.4 (2.6)
BASDAI Q56 (morning stiffness), 0–10, mean (SD)	3.4 (2.8)
Medication use	
NSAID, *n* (%)	93 82%)^a^
ASAS NSAID score, mean (SD)	55.6 (42.6)^b^
bDMARDs, *n* (%)	1 (1%)^a^
csDMARDS, *n* (%)	11 (9%)^b^

a <1% missing data.

b <5% missing data.

c >5% missing data.

Values without a footnote had complete data.

BMI: body mass index; bDMARDS: biological disease-modifying anti-rheumatic drugs; csDMARDS: conventional synthetic disease-modifying anti-rheumatic drugs; ASAS: Assessment of SpondyloArthritis International Society; RDCI: Rheumatic Disease Comorbidity Index; ASDAS: Axial Spondyloarthritis Disease Activity Score; SF36 MCS: Short Form (36) Mental Component Summary score; MEI: Mander Enthesitis Index.

Patients made most use of *comforting cognitions* [mean: 3.0 (SD: 0.5)] to cope with pain, *optimism* [3.1 (0.5)] to cope with limitations and *showing considerations* [2.8 (0.4)] to cope with dependence (Table 2). Moderate correlations were seen between *optimism* and *comforting cognitions* (r = 0.63), *pacing* and *decreasing activities* (r = 0.63) and *creative solution seeking* and *diverting attention* (r = 0.53). All other correlations were low, suggesting that most coping strategies represent distinct constructs (Supplementary Tables S3–S5). However, strategies to cope with pain and limitations, and specifically the most used ones, appeared to be more closely related, indicating potential overlap in these domains and possible higher order coping strategies.

**Table 2:** Descriptives of CORS strategies at both timepoints and association between time and coping strategy scores.

	Initial assessment (*n* = 102)	Two-year follow-up (*n* = 102)	GEE Analysis (*n* = 102) β (95% CI)
Pain			
Comforting cognitions (1–4)	3.0 (0.5)	3.0 (0.5)	−0.04 (−0.13–0.05)
Decreasing activities (1–4)	2.4 (0.5)	2.4 (0.5)	0.0 (−0.10–0.10)
Diverting attention (1–4)	2.4 (0.5)	2.4 (0.6)	−0.02 (−0.12–0.09)
Limitations			
Optimism (1–4)	3.1 (0.5)	3.1 (0.5)	0.07 (−0.03–0.17)
Pacing (1–4)	2.7 (0.6)	2.7 (0.6)	0.01 (−0.06–0.09)
Creative solution seeking (1–4)	2.6 (0.6)	2.6 (0.6)	0.05 (−0.05–0.14)
Dependence			
Accepting dependence (1–4)	2.3 (0.7)	2.3 (0.6)	0.0 (−0.12–0.12)
Showing consideration (1–4)	2.8 (0.4)	2.8 (0.5)	0.03 (−0.05–0.11)

Values are expressed as mean (SD) unless indicated otherwise.

GEE: generalized estimating equations.

### Factors associated with coping strategies

The results of univariable analyses into factors associated with each different coping strategy can be found in Supplementary Table S6. Each coping strategy was associated with one or more patient characteristics, axSpA phenotype and axSpA health score without clear patterns across strategies. Most associations were weak, with education consistently showing an important impact on most coping strategies. Variables not associated with any strategy were IBD, uveitis and HLA-B27 status.

### Coping with pain

To cope with pain, higher education [β for lower *vs* higher education: −0.22 (95%CI: −0.41 to −0.03)] and a higher BMI [0.01 (0.00–0.03)] were associated with more use of *comforting cognitions* in Model A (Table 3). Adding health scores (Model B) indicated that higher SF36-MCS scores [0.01 (0.00–0.02)] were weakly associated with *comforting cognitions* while the association with lower education became non-significant. Further, psoriasis [−0.36 (−0.71 to −0.02)] was negatively associated with *decreasing activities*. When adding health scores (Model B), the coefficient of psoriasis became non-significant, while a significant but numerically small association with more fatigue (BASDAI Q1) [0.04 (0.00–0.09)] was seen. Finally, male sex [−0.21 (−0.38 to −0.04)] and more comorbidities [−0.13 (−0.23 to −0.03)] were negatively associated with *diverting attention* in the main model (Model A). Adding health scores (Model B) did not substantially change the model.

**Table 3:** Associations between stable characteristics and stable plus variable axSpA health scores and pain coping strategies.

	Comforting cognitions, range 1–4	Comforting cognitions, range 1–4	Decreasing activities, range 1–4	Decreasing activities, range 1–4	Diverting attention, range 1–4	Diverting attention, range 1–4
	(*n* = 110)	(*n* = 99)	(*n* = 112)	(*n* = 106)	(*n* = 114)	(*n* = 110)
	Model A	Model B	Model A	Model B	Model A	Model B
	β (95% CI)	β (95% CI)	β (95% CI)	β (95% CI)	β (95% CI)	β (95% CI)
Age, years	0.01 (0.00–0.02)	0.01 (0.00–0.01)	0.00 (−0.01–0.01)	0.01 (0.00–0.01)	0.00 (0.00–0.01)	0.01 (0.00–0.02)
Sex (male *vs* female)	0.08 (−0.09–0.24)	−0.02 (−0.18–0.15)	−0.17 (−0.36–0.03)	−0.07 (−0.28–0.14)	−**0.21 (**−**0.38 to** −**0.04)**	−**0.18 (**−**0.35–0.00)**
Lower *vs* higher education	−**0.22 (**−**0.41 to** −**0.03)**	−0.15 (−0.37–0.07)	0.19 (−0.08–0.46)	0.17 (−0.09–0.44)	0.12 (−0.11–0.34)	0.13 (−0.10–0.35)
BMI, kg/m^2^	**0.01 (0.00–0.03)^a^**	**0.02 (0.00–0.03)** ^a^	^b^	^b^	^b^	^b^
RDCI, 0–9	^b^	^b^	0.03 (−0.07–0.15)^a^	0.04 (−0.08–0.15)^a^	−**0.13 (**−**0.23 to** −**0.03)**^a^	−0.09 (−0.20–0.03)^a^
Currently working (no *vs* yes)	−0.07 (−0.25–0.12)^a^	−0.05 (−0.24–0.15)^a^	0.17 (−0.05–0.38)^a^	0.21 (−0.02–0.43)^a^	^b^	^b^
Disease duration, years	^b^	^b^	^b^	^b^	^b^	^b^
Presence of IBD (ever)	^b^	^b^	^b^	^b^	^b^	^b^
Presence of peripheral arthritis	^b^	^b^	^b^	^b^	^b^	^b^
Presence of uveitis (ever)	^b^	^b^	^b^	^b^	^b^	^b^
Presence of psoriasis (ever)	^b^	^b^	−**0.36 (**−**0.71 to** −**0.02)**^a^	−0.30 (−0.73–0.13)^a^	^b^	^b^
Enthesitis (MEI), 0–90	^b^	^b^	0.00 (0.00–0.01)^a^	0.00 (0.00–0.01)^a^	^b^	^b^
HLA-B27 positive	^b^	^b^	^b^	^b^	^b^	^b^
ASDAS		0.01 (−0.08–0.09)^a^		−0.04 (−0.12–0.05)^a^		^b^
BASFI, 0–10		−0.02 (−0.06–0.02)^a^		−0.01 (−0.06–0.03)^a^		−0.03 (−0.08–0.01)^a^
BASMI, 0–10		^b^		^b^		−0.02 (−0.09–0.04)^a^
BASDAI Q1, 0–10		Not selected^c^		**0.04 (0.00–0.09)** ^a^		^b^
SF36 MCS, 0–100		**0.01 (0.00–0.02)** ^a^		0.00 (−0.01–0.00)^a^		^b^

a Confounded the relationship between one of the three main variables of interest (age, sex, education level) and the outcome in this model.

b In univariable analysis not significant for this strategy, and therefore not included in the multivariable analyses.

c Value not significant and no confounder of the main relationships of interest.

Model A: includes patient characteristics and axSpA phenotypes. Model B: includes patient characteristics, axSpA phenotypes and axSpA health scores.

Bold indicates significance (*P* < 0.05).

RDCI: Rheumatic Disease Comorbidity Index; BMI: body mass index; MEI: Mander Enthesitis Index; SF36 MCS: Short Form (36) Mental Component Summary Score; ASDAS: Axial Spondyloarthritis Disease Activity Score; BASMI: Bath Ankylosing Spondylitis Mobility Index; BASFI: Bath Ankylosing Spondylitis Functional Index; BASDAI: Bath Ankylosing Spondylitis Disease Activity Index.

### Coping with limitations

To cope with limitations, older age [0.02 (0.01–0.03)] was positively associated, while lower education [−0.36 (−0.58 to −0.13)] and comorbidities [−0.11 (−0.20 to −0.02)] were negatively associated with *optimism* in Model A (Table 4). Including health scores (Model B) slightly attenuated the association with education [−0.28 (−0.49 to −0.07)] while higher SF36-MCS scores [0.01 (0.00–0.02)] showed a significant but numerically small association. For *pacing*, a significant interaction between work status and education level was found (*P* = 0.049). However, stratification did not result in clinically relevant differences between the groups; therefore, the final model was presented for the overall population. Older age [0.01 (0.01–0.02)] was associated with *pacing*, and the addition of health scores (Model B) did not change the models considerably. For *creative solution seeking*, no variables were found to be significantly associated in any of the models.

**Table 4:** Associations between stable characteristics and stable plus variable axSpA health scores and limitations coping strategies.

	Optimism, range 1–4	Optimism, range 1–4	Pacing, range 1–4	Pacing, range 1–4	Creative solution seeking, range 1–4	Creative solution seeking, range 1–4
	(*n* = 110)	(*n* = 104)	(*n* = 114)	(*n* = 109)	(*n* = 114)	(*n* = 113)
	Model A	Model B	Model A	Model B	Model A	Model B
	β (95% CI)	β (95% CI)	β (95% CI)	β (95% CI)	β (95% CI)	β (95% CI)
Age, years	**0.02 (0.01–0.03)**	**0.02 (0.01–0.03)**	**0.01 (0.01–0.02)**	**0.01 (0.00–0.02)**	0.01 (0.00–0.01)	0.01 (0.00–0.01)
Sex (male *vs* female)	0.08 (−0.10–0.27)	−0.05 (−0.26–0.16)	−0.14 (−0.35–0.07)	−0.13 (−0.35–0.10)	−0.13 (−0.33–0.07)	−0.14 (−0.33–0.6)
Lower *vs* higher education	−**0.36 (**−**0.58 to** −**0.13)**	−**0.28 (**−**0.49 to** −**0.07)**	0.11 (−0.19–0.40)	0.06 (−0.24–0.35)	−0.03 (−0.32–0.26)	−0.04 (−0.33–0.25)
BMI, kg/m^2^	^b^	^b^	^b^	^b^	^b^	^b^
RDCI, 0–9	−**0.11 (**−**0.20 to** −**0.02)**	−**0.09 (−0.17 to** −**0.02)**	0.05 (−0.07–0.16)^a^	0.02 (−0.10–0.14)^a^	^b^	^b^
Currently working (no *vs* yes)	^b^	^b^	0.15 (−0.10–0.41)^a^	0.13 (−0.13–0.39)^a^	0.15 (−0.09–0.39)^a^	0.13 (−0.13–0.38)^a^
Disease duration, years	0.00 (−0.01–0.01)^a^	−0.01 (−0.01–0.00)^a^	^b^	^b^	^b^	^b^
Presence of IBD (ever)	^b^	^b^	^b^	^b^	^b^	^b^
Presence of peripheral arthritis	^b^	^b^	^b^	^b^	^b^	^b^
Presence of uveitis (ever)	^b^	^b^	^b^	^b^	^b^	^b^
Presence of psoriasis (ever)	^b^	^b^	^b^	^b^	^b^	^b^
Enthesitis (MEI), 0–90	^b^	^b^	^b^	^b^	^b^	^b^
HLA-B27 positive	^b^	^b^	^b^	^b^	^b^	^b^
ASDAS		−0.04 (−0.13–0.06)^a^		0.05 (−0.03–0.13)^a^		^b^
BASFI, 0–10		^b^		−0.01 (−0.06–0.04)^a^		0.01 (−0.03–0.05)^a^
BASMI, 0–10		^b^		0.05 (−0.02–0.05)^a^		^b^
BASDAI Q1, 0–10		−0.01 (−0.04–0.03)^a^		0.02 (−0.01–0.05)^a^		^b^
SF36 MCS, 0–100		**0.01 (0.00–0.02)** ^a^		^b^		^b^

a Confounded the relationship between one of the three main variables of interest (age, sex, education level) and the outcome in this model.

b In univariable analysis not significant for this strategy, and therefore not included in the multivariable analyses.

Model A: includes patient characteristics and axSpA phenotypes. Model B: includes patient characteristics, axSpA phenotypes and axSpA health scores.

Bold indicates significance (*P* < 0.05).

RDCI: Rheumatic Disease Comorbidity Index; BMI: body mass index; MEI: Mander Enthesitis Index; SF36 MCS: Short Form (36) Mental Component Summary Score; ASDAS: Axial Spondyloarthritis Disease Activity Score; BASMI: Bath Ankylosing Spondylitis Mobility Index; BASFI: Bath Ankylosing Spondylitis Functional Index; BASDAI: Bath Ankylosing Spondylitis Disease Activity Index.

### Coping with dependence

To cope with dependence, psoriasis [−0.28 (−0.49 to −0.07)] was negatively and enthesitis (MEI) [0.01 (0.00–0.01)] was positively associated with *accepting dependence* (Table 5). Health scores (model B) were not associated with this specific coping strategy, but their inclusion made the associations of psoriasis and enthesitis non-significant. For *showing consideration*, a statistically significant interaction was found between comorbidities and education level (*P* < 0.001). Among lower educated patients, comorbidities were negatively associated [−0.12 (−0.21 to −0.02)], while among higher educated patients, comorbidities were positively associated [1.14 (0.08–2.20)] with *showing consideration*. However, due to the limited number of higher-educated patients (*n* = 20 in this specific model) this stratified analysis was mostly exploratory. Adding axSpA health scores to the (non-stratified) model (Model B) did not result in relevant changes.

**Table 5:** Associations between stable characteristics and stable plus variable axSpA health scores and dependence coping strategies.

	Accepting dependence, range 1–4	Accepting dependence, range 1–4	Showing consideration, range 1–4	Showing consideration, range 1–4
	(*n* = 112)	(*n* = 107)	(*n* = 113)	(*n* = 113)
	Model A	Model B	Model A	Model B
	β (95% CI)	β (95% CI)	β (95% CI)	β (95% CI)
Age, years	0.01 (0.00–0.02)	0.01 (0.00–0.02)	0.00 (0.00–0.01)	0.00 (0.00–0.01)
Sex (male)	−0.03 (−0.25–0.19)	−0.14 (−0.36–0.08)	−0.14 (−0.30–0.02)	−0.14 (−0.30–0.02)
Lower *vs* higher education	0.13 (−0.21–0.46)	0.04 (−0.32–0.39)	−0.09 (−0.32–0.14)	−0.10 (−0.32–0.14)
BMI, kg/m^2^	^b^	^b^	^b^	^b^
RDCI, 0–9	0.01 (−0.18–0.20)^a^	−0.02 (−0.20–0.16)^a^	−**0.10 (−0.19–0.00)**^a^	−**0.10 (−0.19–0.00)**^a^
Currently working (no *vs* yes)	0.12 (−0.16–0.39)^a^	0.04 (−0.21–0.30)^a^	^b^	^b^
Disease duration, years	^b^	^b^	^b^	^b^
Presence of IBD (ever)	^b^	^b^	^b^	^b^
Presence of peripheral arthritis	^b^	^b^	−0.13 (−0.27–0.00)^a^	−0.13 (−0.27–0.00)^a^
Presence of uveitis (ever)	^b^	^b^	^b^	^b^
Presence of psoriasis (ever)	−**0.28 (−0.49 to** −**0.07)**^a^	−0.28 (−0.57–0.01)^a^	−0.20 (−0.44–0.04)^a^	−0.20 (−0.44–0.04)^a^
Enthesitis (MEI), 0–90	**0.01 (0.00–0.01)** ^a^	0.00 (0.00–0.01)^a^	0.00 (0.00–0.01)^a^	0.00 (0.00–0.01)^a^
HLA-B27 positive	^b^	^b^	^b^	^b^
ASDAS		0.06 (−0.04–0.16)^a^		^b^
BASFI, 0–10		0.02 (−0.04–0.08)^a^		^b^
BASMI, 0–10		0.05 (0.03–0.12)^a^		^b^
BASDAI Q1, 0–10		Not selected^c^		^b^
SF36 MCS, 0–100		0.00 (−0.01–0.00)^a^		^b^

a Confounded the relationship between one of the three main variables of interest (age, sex, education level) and the outcome in this model.

b In univariable analysis not significant for this strategy, and therefore not included in the multivariable analyses.

c Value not significant and no confounder of the main relationships of interest.

Model A: includes patient characteristics and axSpA phenotypes. Model B: includes patient characteristics, axSpA phenotypes and axSpA health scores.

Bold indicates significance (*P* < 0.05).

RDCI: Rheumatic Disease Comorbidity Index; BMI: body mass index; MEI: Mander Enthesitis Index; SF36 MCS: Short Form (36) Mental Component Summary Score; ASDAS: Axial Spondyloarthritis Disease Activity Score; BASMI: Bath Ankylosing Spondylitis Mobility Index; BASFI: Bath Ankylosing Spondylitis Functional Index; BASDAI: Bath Ankylosing Spondylitis Disease Activity Index.

In general, when replacing ASDAS by individual components of disease activity (patient global assessment, back pain and morning stiffness) no substantial changes were seen compared with the models with ASDAS (Models C *vs* Models B) (Supplementary Table S7).

### Change in coping strategies over time

In total, 102 patients had data on coping strategies at both time points. Descriptive statistics indicated no changes over time across all strategies (Table 2). GEE analyses showed no significant effect of time on coping strategy scores either, with coefficients reflecting the use of strategies over time ranging from −0.04 to 0.07 per two years.

## Discussion

Patients with long-standing axSpA make use of a variety of cognitive and behavioural coping strategies to deal with disease-related stressors. In our study, coping strategies remained stable over two years, with only a few and at times no predictors being associated with the use of each of the various strategies in multivariable analyses. No single factor was associated with all strategies, and relatively weak associations were found. Overall, coping was more dependent on stable socio-demographic factors than on (stable) general health/axSpA phenotypes or variable health scores. Although the literature makes a clear distinction between cognitive and behavioural coping strategies, we could not find strong differences between both types of strategies in frequency of use, stability over time or associated factors between these larger styles.

The limited number of associations in the multivariable models contrasted with the larger number of associations in the univariable analyses. Partly this is due to the limited sample size, but partly also due to confounding between socio-demographic factors or disease phenotypes and health scores [27]. Moreover, axSpA health scores had a negligible or low association with the use of coping strategies in the final models, indicating that coping use is not situationally dependent on current disease symptoms.

As for the more stable socio-demographic factors, a higher level of education had a relatively strong association with coping with limitations by *optimism* in multivariable analyses. It seems plausible that persons with a higher education have stronger self-efficacy skills and more resources to maintain control over the disease and thereby adopt a more optimistic coping style. Also, female sex showed a (relatively weak) association with *diverting attention* to cope with pain and higher age with *pacing* and *optimism* to cope with limitations. Although previous research confirmed sex differences in coping and an association between higher age and adapting activities as a coping style, we should be careful not to over-interpret the relatively weak findings in a setting where multiple variables are tested [14].

Another finding that merits discussion is the association of the SF36-MCS with *comforting cognitions* to cope with pain and *optimism* to cope with limitations – two cognitive strategies – that seem kind of related. Better mental health likely indicates a more satisfied and at-ease state of mind, attenuating negative thoughts. It could be questioned whether SF-36 MCS perhaps also reflects a personal trait in addition to measuring true current mental health. Along the same line, the link between fatigue and *decreasing activities* as a way to cope with pain was not surprising, and the association between fewer comorbidities and *optimism* to cope with limitations is intuitive, but the magnitude of the association is small.

To our knowledge, only one study previously assessed factors associated with coping strategies [15]. This study reported that female sex and worse physical function were associated with *decreasing activities*. Although we observed such associations in univariable analyses, they did not persist in the multivariable models. In the earlier study, older age was also associated with *pacing*, but female sex and worse physical function as well. Contradicting findings may reflect methodological differences and the complexity of the construct of coping. Our study’s larger, more diverse sample, with two timepoints, and broader inclusion of potentially explanatory characteristics in the models likely enabled more robust and less confounded associations.

Our findings regarding change over time are in line with analyses of the two-year follow-up of the SPACE axSpA inception cohort and the four-year follow-up of the Dutch standard diagnosis register of rheumatic diseases (SDR), where it was demonstrated that no association between disease duration and coping strategies existed and where coping strategies remained stable over time in patients with early as well as in those with established axSpA [15, 16]. This observed stability of coping strategies suggests that they are unlikely to change without targeted intervention. This underscores the importance of psychosocial assessment and potentially, coping-focused interventions, such as cognitive behavioural therapy [28]. It should be noted that the current study and the inception cohort study analysed change at the group level. The SDR study, which evaluated *pacing* to cope with limitations and *decreasing activities* to cope with pain, analysed individual change and found only moderate agreement in coping strategy scores at baseline and four-year follow-up (ICC = 0.70 and 0.56, respectively) [15].

This study has several limitations. First, selection bias cannot be excluded because of a substantial proportion of dropouts at 4 years of follow-up of the OASIS cohort. Although included and excluded participants were largely comparable, they differed in education level, which may limit the generalizability, particularly for the higher educated. Second, the population, though valuable for its longitudinal nature, was not sufficiently large to enable robust assessment of interaction effects. Lastly, although a broad range of patient and disease-specific variables was included, there was limited information on psychosocial and personality traits and factors like race and ethnicity or other cultural background descriptors, which hampered the evaluation of these factors in the development of coping strategies. Although coping is often suggested as a relevant personal factor influencing health outcomes, few instruments are available. We feel the CORS is a promising instrument as it was developed with strong involvement of patients [6]. Overall, the correlations between strategies were low and there was little overlap in explanatory variables indicating that they represent distinct aspects of coping. A challenge in assessing its validity is the absence of appropriate external standards to test hypothesis on construct validity. A key strength of this study is the use of data from the well-established, longitudinal and multinational OASIS cohort.

While the lack of observed change in coping strategies over time and the limited associations found with variable factors in this study support the assumption of their relative stability, more research is needed. These studies should focus on individual differences, their magnitude and meaning, to further clarify coping change over time. Measuring illness perceptions in addition to more extensive patient characteristics would also provide insight into how these perceptions guide the formation of coping strategies as they have been suggested to be precursors to coping strategies [9, 29, 30]. Finally, studies with larger sample sizes are necessary to enable more detailed subgroup analyses. A logical next question, now that more is known about which individuals use which coping strategies, would be to determine how the use of these strategies influences disease outcomes. This could help identify specific subgroups of patients for whom targeted interventions may be needed to improve outcomes.

In conclusion, coping strategies in axSpA appear more strongly influenced by patient characteristics, to a lesser extent by axSpA phenotypes and not by axSpA-related health scores. Furthermore, no change over time was found supporting that coping is a trait and more dependent on the person than the disease. Future studies should explore whether and how these coping strategies impact long-term outcomes to better identify patients who may benefit from tailored interventions.

## Supplementary Material

keag140_Supplementary_Data

## Data Availability

The data underlying this article were provided by Maastricht University by permission. Data will be shared upon a formal proposal submitted to the corresponding author with permission of Maastricht University.
