# Supplementary material for: Coping with rheumatic stressors in long-standing axial spondyloarthritis: association with patient and disease characteristics
Source: Rheumatology (Oxford). 2026 Mar 25;65(4):keag140. doi: 10.1093/rheumatology/keag140 (PMC13070635; doi:10.1093/rheumatology/keag140)
Supplement: keag140_Supplementary_Data [file keag140_supplementary_data.docx]

**Title: Coping with Rheumatic Stressors in longstanding Axial Spondyloarthritis: Association with Patient and Disease Characteristics**

Authors: Marc van Essen, Dafne Capelusnik, Désirée van der Heijde, Robert Landewé, Wim van Lankveld, Astrid van Tubergen, Sofia Ramiro, Annelies Boonen

**Supplementary Table S1: Example questions for each subscale of the CORS questionnaire**

| **CORS domain** | **CORS subscale** | **Cognitive or behavioural** | **Example items** |
| --- | --- | --- | --- |
| Coping with pain | Comforting cognitions | Cognitive | “I think the pain will become better”  “I try to enjoy myself despite the pain” |
|  | Decreasing activities | Behavioural | “I limit my activities to easy tasks”  “I take a rest by sitting or lying down” |
|  | Diverting attention | Cognitive | “I think about pleasant things or happenings” “I take up a task in which I will not feel the pain” |
| Coping with limitations | Optimism | Cognitive | “I’m happy with what I can do”  “I try to remain optimistic” |
|  | Pacing | Behavioural | “I take my limitations into account”  “I let others do the work” |
|  | Creative solution seeking | Cognitive | “I think of solutions for my limitations”  “I think of new ways to handle the tasks” |
| Coping with dependence | Accepting dependence | Cognitive | “I try to accept my dependence”  “I accept my dependence” |
|  | Showing consideration | Cognitive | “I try to take others into account”  “I try to do something in return” |

**Supplementary Table S2: Comparison of baseline characteristics (entry in OASIS) of patients with and without COping with Rheumatic Stressors (CORS) at the fourth year if follow-up**

| **Assessment** | **CORS available at year 4**  **N=116** | **No CORS available at your 4**  **N=101** |
| --- | --- | --- |
| Age, years, mean (SD) | 44.8 (11.6) | 42.1 (13.7) |
| Sex, male, n (%) | 84 (72%) | 70 (69%) |
| Education level   - Lower, n (%) - Higher, n (%) | 94 (82%) ^a^  21 (18%) ^a^ | 62 (61%) ^b^  36 (36%) ^b^ |
| BMI, kg/m^2^, mean (SD) | 25.2 (4.6) | 24.1 (3.5) |
| RDCI, 0-9, mean (SD) | 0.3 (0.8) | 0.0 (0.3) |
| Currently working, >33hr/week, n (%) | 48 (42%) ^a^ | 55 (54%) |
| Duration of symptoms, years, mean (SD) | 22.2 (11.6) ^b^ | 18.3 (11.6) |
| Presence of IBD, ever, n (%) | 12 (10%) ^a^ | 3 (3%) |
| Presence of peripheral arthritis, current, n (%) | 28 (24%) | 28 (28%) |
| Presence of Uveitis, ever, n (%) | 21 (18%) ^a^ | 18 (18%) |
| Presence of Psoriasis, ever, n (%) | 5 (4%) ^a^ | 4 (4%) |
| Enthesitis (MEI), 0-90, mean (SD) | 12.2 (16.5) | 8.8 (14.4) |
| HLA-B27 positive, n (%) | 89 (80%) ^b^ | 85 (89%) ^b^ |
| ASDAS, mean (SD) | 2.7 (0.9) ^c^ | 2.8 (1.2) ^b^ |
| BASFI, 0-10, mean (SD) | 3.7 (2.5) ^c^ | 3.1 (2.7) ^b^ |
| BASMI, 0-10, mean (SD) | 3.8 (1.5) ^c^ | 3.8 (1.8) ^b^ |
| BASDAI Q1 (fatigue), 0-10, mean (SD) | 4.5 (2.9) ^b^ | 4.4 (2.9) |
| SF36 MCS, 0-100, mean (SD) | n.a. ^d^ | n.a. ^d^ |
| BASDAI Q2 (back pain), 0-10, mean (SD) | 4.3 (2.6) | 4.3 (2.9) |
| Patient global assessment, 0-10, mean (SD) | 3.4 (2.5) ^a^ | 4.2 (3.1) |
| BASDAI Q56 (morning stiffness), 0-10, mean (SD) | 3.5 (2.6) | 3.6 (2.8) |
| Medication use   - NSAID, n (%) - ASAS NSAID score, mean (SD) - bDMARDs, n (%) - csDMARDS, n (%) | 71 (61%)  47.3 (49.2)  0 (0%)  8 (7%) | 75 (74%)  69.7 (57.0)  0 (0%)  10 (10%) |

^a^ <1% missing data;

^b^ <5% missing data;

^c^ >5% missing data;

^d^ Measurements were not performed at month 0.

BMI: Body Mass Index; bDMARDS: Biological Disease-Modifying Anti-Rheumatic Drugs; csDMARDS: Conventional synthetic Disease-Modifying Anti-Rheumatic Drugs; ASAS: Assessment of SpondyloArthritis International Society; RDCI: Rheumatic Disease Comorbidity Index; ASDAS: Axial Spondyloarthritis Disease Activity Score; SF36 MCS: Short Form (36) Mental Component Summary score; MEI: Mander Enthesitis Index.

**Supplementary Table S3: Correlation between coping with pain strategies and coping with limitations strategies**

|  | | Pain | | |
| --- | --- | --- | --- | --- |
|  |  | Comforting cognitions | Decreasing activities | Diverting attention |
| Limitations | Optimism | 0.63 | -0.15 | 0.24 |
|  | Pacing | -0.35 | 0.63 | 0.22 |
|  | Creative solution seeking | 0.21 | 0.38 | 0.51 |

**Supplementary Table S4: Correlation between coping with pain strategies and coping with dependence strategies**

|  | | Pain | | |
| --- | --- | --- | --- | --- |
|  |  | Comforting cognitions | Decreasing activities | Diverting attention |
| Dependence | Accepting dependence | 0.02 | 0.24 | 0.02 |
|  | Showing consideration | 0.24 | 0.14 | 0.33 |

**Supplementary Table S5: Correlation between coping with limitations strategies and coping with dependence strategies**

|  | | Limitations | | |
| --- | --- | --- | --- | --- |
|  |  | Optimism | Pacing | Creative solution seeking |
| Dependence | Accepting dependence | 0.15 | 0.39 | 0.21 |
|  | Showing consideration | 0.28 | 0.19 | 0.44 |

**Supplementary Table S6: Associations between stable patient characteristics, axSpA phenotypes, variable axSpA health scores and each of the eight coping strategies (univariable models)**

|  | Comforting cognitions, range 1-4  n=112-116 | Decreasing activities, range 1-4  n=112-116 | Diverting attention, range 1-4  n=111-115 | Optimism, range 1-4  n=112-116 | Pacing, range 1-4  n=112-116 | Creative solution seeking, range 1-4  n=112-116 | Accepting dependence, range 1-4  n=112-116 | Showing consideration, range 1-4  n=112-116 |
| --- | --- | --- | --- | --- | --- | --- | --- | --- |
|  | β (95% CI) | β (95% CI) | β (95% CI) | β (95% CI) | β (95% CI) | β (95% CI) | β (95% CI) | β (95% CI) |
| Age, years | 0.00 (-0.00 to 0.01) | **0.01 (0.00 to 0.01)** | 0.00 (-0.00 to 0.01) | **0.02 (0.01 to 0.02)** | **0.02 (0.01 to 0.03)** | **0.01 (0.0 to 0.02)** | **0.01 (0.00 to 0.02)** | 0.00 (-0.01 to 0.01) |
| Sex (male vs female) | 0.05 (-0.12 to 0.22) | **-0.26 (-0.44 to 0.09)** | **-0.22 (-0.40 to -0.04)** | 0.03 (-0.18 to 0.23) | **-0.20 (-0.42 to 0.03)** | **-0.19 (-0.38 to 0.00)** | **-0.16 (-0.38 to 0.07)** | **-0.16 (-0.32 to 0.00)** |
| Lower vs higher education | **-0.27 (-0.43 to -0.10)** | **0.30 (0.04 to 0.57)** | 0.08 (-0.15 to 0.31) | **-0.36 (-0.59 to -0.13)** | **0.29 (0.00 to 0.58)** | 0.06 (-0.20 to 0.31) | **0.25 (-0.03 to 0.53)** | -0.08 (-0.30 to 0.14) |
| BMI, kg/m^2^ | **0.02 (0.00 to 0.03)** | 0.01 (-0.01 to 0.02) | -0.01 (-0.02 to 0.01) | 0.01 (-0.01 to 0.03) | 0.00 (-0.02 to 0.02) | 0.00 (-0.02 to 0.02) | 0.01 (-0.01 to 0.03) | 0.00 (-0.01 to 0.01) |
| RDCI, 0-9 | -0.02 (-0.10 to 0.07) | **0.12 (0.02 to 0.22)** | **-0.09 (-0.19 to 0.01)** | **-0.07 (-0.16 to 0.02)** | **0.15 (0.06 to 0.25)** | 0.04 (-0.04 to 0.12) | **0.11 (-0.05 to 0.28)** | **-0.08 (-0.17 to 0.01)** |
| Currently working (no vs yes) | **-0.13 (-0.28 to 0.03)** | **0.31 (0.14 to 0.48)** | 0.10 (-0.07 to 0.28) | -0.05 (-0.23 to 0.14) | **0.32 (0.12 to 0.53)** | **0.21 (0.02 to 0.41)** | **0.30 (0.09 to 0.51)** | 0.05 (-0.11 to 0.22) |
| Disease duration, years | 0.00 (-0.03 to 0.01) | 0.00 (-0.01 to 0.01) | 0.00 (-0.01 to 0.01) | **0.01 (0.00 to 0.02)** | 0.01 (-0.01 to 0.02) | 0.00 (-0.01 to 0.01) | 0.00 (-0.01 to 0.02) | 0.00 (-0.01 to 0.01) |
| Presence of IBD (ever) | 0.13 (-0.14 to 0.40) | -0.07 (-0.36 to 0.22) | 0.12 (-0.16 to 0.40) | -0.02 (-0.30 to 0.26) | -0.10 (-0.41 to 0.21) | -0.13 (-0.43 to 0.16) | 0.09 (-0.20 to 0.38) | 0.07 (-0.18 to 0.31) |
| Presence of peripheral arthritis | 0.09 (-0.06 to 0.24) | -0.11 (-0.29 to 0.07) | 0.04 (-0.16 to 0.24) | 0.10 (-0.09 to 0.30) | -0.05 (-0.22 to 0.16) | -0.02 (-0.17 to 0.14) | -0.01 (-0.24 to 0.21) | **-0.11 (-0.24 to 0.02)** |
| Presence of uveitis (ever) | 0.10 (-0.09 to 0.29) | -0.06 (-0.25 to 0.14) | 0.02 (-0.18 to 0.23) | -0.01 (-0.21 to 0.20) | -0.10 (-0.32 to 0.12) | 0.00 (-0.22 to 0.22) | 0.01 (-0.23 to 0.25) | 0.00 (-0.17 to 0.17) |
| Presence of psoriasis (ever) | -0.26 (-0.81 to 0.29) | **-0.31 (-0.68 to 0.06)** | -0.10 (-0.34 to 0.13) | -0.18 (-0.51 to 0.15) | 0.00 (-0.23 to 0.24) | -0.07 (-0.22 to 0.08) | **-0.20 (-0.44 to 0.04)** | **-0.15 (-0.36 to 0.06)** |
| Enthesitis (MEI), 0-90 | 0.00 (-0.01 to 0.00) | **0.01 (0.00 to 0.01)** | 0.00 (0.00 to 0.01) | 0.00 (-0.01 to 0.00) | 0.00 (0.00 to 0.01) | 0.00 (0.00 to 0.01) | **0.01 (0.00 to 0.01)** | **0.00 (0.00 to 0.01)** |
| HLA-B27 positive | 0.03 (-0.17 to 0.22) | -0.02 (-0.22 to 0.18) | -0.12 (-0.35 to 0.11) | 0.03 (-0.18 to 0.25) | -0.13 (-0.35 to 0.09) | -0.08 (-0.31 to 0.16) | -0.05 (-0.31 to 0.21) | -0.11 (-0.27 to 0.06) |
| ASDAS | **-0.05 (-0.11 to 0.02)** | **0.06 (-0.02 to 0.13)** | -0.04 (-0.11 to 0.03) | **-0.08 (-0.16 to 0.00)** | **0.08 (0.02 to 0.14)** | 0.03 (-0.04 to 0.10) | **0.12 (0.04 to 0.19)** | 0.01 (-0.04 to 0.07) |
| BASFI, 0-10 | **-0.03 (-0.06 to 0.01)** | **0.04 (0.01 to 0.07)** | **-0.04 (-0.07 to 0.00)** | -0.02 (-0.05 to 0.02) | **0.06 (0.02 to 0.09)** | **0.02 (-0.01 to 0.06)** | **0.08 (0.05 to 0.11)** | 0.01 (-0.02 to 0.04) |
| BASMI, 0-10 | 0.00 (-0.04 to 0.05) | 0.02 (-0.04 to 0.07) | **-0.05 (-0.10 to 0.00)** | 0.02 (-0.03 to 0.07) | **0.07 (0.02 to 0.12)** | 0.03 (-0.03 to 0.09) | **0.10 (0.04 to 0.15)** | 0.00 (-0.04 to 0.04) |
| BASDAI Q1, 0-10 | **-0.02 (-0.05 to 0.00)** | **0.05 (0.00 to 0.08)** | -0.02 (-0.04 to 0.01) | **-0.03 (-0.06 to 0.00)** | **0.05 (0.02 to 0.07)** | 0.01 (-0.01 to 0.04) | **0.03 (0.00 to 0.07)** | 0.01 (-0.01 to 0.03) |
| SF36 MCS, 0-100 | **0.01 (0.00 to 0.01)** | **-0.01 (-0.01 to 0.00)** | 0.00 (-0.01 to 0.01) | **0.01 (0.00 to 0.02)** | 0.00 (0.00 to 0.01) | 0.00 (-0.01 to 0.01) | **-0.01 (-0.01 to 0.00)** | 0.00 (-0.01 to 0.00) |
| BASDAI Q2, 0-10 | **-0.02 (-0.05 to 0.01)** | **0.04 (0.02 to 0.07)** | -0.01 (-0.04 to 0.02) | **-0.03 (-0.06 to 0.00)** | **0.04 (0.01 to 0.06)** | 0.02 (-0.01 to 0.04) | **0.05 (0.01 to 0.08)** | 0.00 (-0.02 to 0.03) |
| Patient global assessment, 0-10 | **-0.03 (-0.06 to 0.00)** | **0.03 (0.00 to 0.06)** | 0.00 (-0.03 to 0.03) | **-0.04 (-0.07 to -0.01)** | **0.02 (0.00 to 0.05)** | 0.01 (-0.02 to 0.04) | 0.01 (-0.02 to 0.04) | 0.01 (-0.01 to 0.03) |
| BASDAI Q56, 0-10 | **-0.03 (-0.06 to 0.00)** | **0.03 (0.00 to 0.05)** | **-0.03 (-0.05 to 0.00)** | **-0.04 (-0.08 to -0.01)** | **0.03 (0.01 to 0.06)** | 0.01 (-0.02 to 0.04) | **0.03 (0.01 to 0.06)** | -0.01 (-0.04 to 0.01) |

Bold indicates significance (p<0.20).

RDCI: Rheumatic Disease Comorbidity Index; BMI: Body Mass Index; SF36 MCS: Short Form (36) Mental Component Summary score; ASDAS: Axial Spondyloarthritis Disease Activity Score; MEI: Mander Enthesitis Index.

**Supplementary Table S7: Associations between patient characteristics (including axSpA phenotypes) and alternative axSpA health scores* and each of the eight coping strategies**

|  | Comforting cognitions, range 1-4  (n=107) | Decreasing activities, range 1-4  (n=109) | Diverting attention, range 1-4  (n=110) | Optimism, range 1-4  (n=109) | Pacing, range 1-4  (n=111) | Creative solution seeking, range 1-4  (n=113) | Accepting dependence, range 1-4  (n=110) | Showing consideration, range 1-4  (n=113) |
| --- | --- | --- | --- | --- | --- | --- | --- | --- |
|  | Model C  β (95% CI) | Model C  β (95% CI) | Model C  β (95% CI) | Model C  β (95% CI) | Model C  β (95% CI) | Model C  β (95% CI) | Model C  β (95% CI) | Model C  β (95% CI) |
| Age, years | 0.01 (0.00 to 0.01) | 0.00 (0.00 to 0.01) | 0.01 (0.00 to 0.02) | **0.02 (0.01 to 0.03)** | **0.01 (0.00 to 0.02)** | 0.01 (0.00 to 0.01) | 0.01 (-0.01 to 0.02) | 0.00 (0.00 to 0.01) |
| Sex (male vs female) | 0.02 (-0.16 to 0.19) | -0.08 (-0.30 to 0.12) | **-0.18 (-0.35 to 0.00)** | -0.02 (-0.22 to 0.18) | -0.11 (-0.34 to 0.12) | -0.14 (-0.33 to 0.06) | -0.07 (-0.30 to 0.16) | -0.14 (-0.30 to 0.02) |
| Lower vs higher education | **-0.21 (-0.40 to -0.01)** | 0.13 (-0.14 to 0.39) | 0.13 (-0.10 to 0.35) | **-0.25 (-0.46 to -0.04)** | 0.04 (-0.25 to 0.32) | -0.04 (-0.33 to 0.25) | 0.09 (-0.25 to 0.44) | -0.10 (-0.32 to 0.14) |
| BMI, kg/m^2^ | 0.01 (0.00 to 0.03) ^a^ | ^b^ | ^b^ | ^b^ | ^b^ | ^b^ | ^b^ | ^b^ |
| RDCI, 0-9 | ^b^ | 0.05 (-0.06 to 0.16) ^a^ | -0.09 (-0.20 to 0.03) ^a^ | **-0.10 (-0.19 to -0.02)** | 0.02 (-0.10 to 0.14) ^a^ | ^b^ | -0.02 (-0.20 to 0.16) ^a^ | **-0.10 (-0.19 to 0.00)** ^a^ |
| Currently working (no vs yes) | -0.02 (-0.21 to 0.16) ^a^ | 0.19 (-0.03 to 0.40) ^a^ | ^b^ | ^b^ | 0.09 (-0.16 to 0.35) ^a^ | 0.13 (-0.13 to 0.38) ^a^ | 0.04 (-0.21 to 0.30) ^a^ | ^b^ |
| Disease duration, years | ^b^ | ^b^ | ^b^ | 0.00 (-0.01 to 0.01)^a^ | ^b^ | ^b^ | ^b^ | ^b^ |
| Presence of IBD (ever) | ^b^ | ^b^ | ^b^ | ^b^ | ^b^ | ^b^ | ^b^ | ^b^ |
| Presence of peripheral arthritis | ^b^ | ^b^ | ^b^ | ^b^ | ^b^ | ^b^ | ^b^ | -0.13 (-0.27 to 0.00) ^a^ |
| Presence of uveitis (ever) | ^b^ | ^b^ | ^b^ | ^b^ | ^b^ | ^b^ | ^b^ | ^b^ |
| Presence of psoriasis (ever) | ^b^ | 0.28 (-0.13 to 0.68) ^a^ | ^b^ | ^b^ | ^b^ | ^b^ | -0.27 (-0.55 to 0.01) ^a^ | -0.20 (-0.44 to 0.04) ^a^ |
| Enthesitis (MEI), 0-90 | ^b^ | 0.00 (0.00 to 0.01) ^a^ | ^b^ | ^b^ | ^b^ | ^b^ | 0.00 (0.00 to 0.01) ^a^ | 0.00 (0.00 to 0.01) ^a^ |
| HLA-B27 positive | ^b^ | ^b^ | ^b^ | ^b^ | ^b^ | ^b^ | ^b^ | ^b^ |
| BASFI, 0-10 | -0.02 (-0.06 to 0.03) ^a^ | -0.02 (-0.07 to 0.03) ^a^ | -0.03 (-0.08 to 0.01) ^a^ | ^b^ | 0.02 (-0.04 to 0.08) ^a^ | 0.01 (-0.03 to 0.05) ^a^ | 0.02 (-0.04 to 0.08) ^a^ | ^b^ |
| BASMI, 0-10 | ^b^ | ^b^ | -0.02 (-0.09 to 0.04) ^a^ | ^b^ | 0.01 (-0.05 to 0.07) ^a^ | ^b^ | 0.06 (-0.01 to 0.12) ^a^ | ^b^ |
| BASDAI Q1, 0-10 | Not selected ^c^ | 0.03 (0.00 to 0.07) ^a^ | ^b^ | 0.01 (-0.03 to 0.04) ^a^ | **0.03 (0.00 to 0.06)** ^a^ | ^b^ | Not selected ^c^ | ^b^ |
| SF36 MCS, 0-100 | **0.01 (0.00 to 0.02)** ^a^ | 0.00 (-0.01 to 0.00) ^a^ | ^b^ | **0.01 (0.00 to 0.02)** ^a^ | ^b^ | ^b^ | 0.00 (-0.01 to 0.00) ^a^ | ^b^ |
| BASDAI Q2, 0-10 | 0.02 (-0.02 to 0.07) ^a^ | 0.03 (-0.02 to 0.07) ^a^ | ^b^ | Not selected ^c^ | Not selected ^c^ | ^b^ | 0.01 (-0.03 to 0.06) ^a^ | ^b^ |
| Patient global assessment, 0-10 | -0.03 (-0.07 to 0.02) ^a^ | Not selected ^c^ | ^b^ | -0.02 (-0.05 to 0.01) ^a^ | -0.01 (-0.04 to 0.02) ^a^ | ^b^ | ^b^ | ^b^ |
| BASDAI Q56, 0-10 | -0.01 (-0.05 to 0.04) ^a^ | -0.02 (-0.06 to 0.01) ^a^ | Not selected ^c^ | -0.02 (-0.06 to 0.03) ^a^ | 0.01 (-0.02 to 0.04) ^a^ | ^b^ | Not selected ^c^ | ^b^ |

* Model C: includes patient characteristics, axSpA phenotypes and alternative axSpA health scores (instead of ASDAS the following were included: BASDAI Q2, patient global assessment and BASDAI Q56)

^a^ Confounded the relationship between one of the three main variables of interest (age, sex, education level) and the outcome in this model;

^b^ In univariable analysis not significant for this strategy, and therefore not included in the multivariable analyses;

^c^ Value not significant and no confounder of the main relationships of interest.

Bold indicates significance (p<0.05).

RDCI: Rheumatic Disease Comorbidity Index; BMI: Body Mass Index; SF36 MCS: Short Form (36) Mental Component Summary score; MEI: Mander Enthesitis Index.
